# Neutrophil-Mediated Regulation of Innate and Adaptive Immunity: The Role of Myeloperoxidase

**DOI:** 10.1155/2016/2349817

**Published:** 2016-01-20

**Authors:** Dragana Odobasic, A. Richard Kitching, Stephen R. Holdsworth

**Affiliations:** ^1^Centre for Inflammatory Diseases, Monash University, Department of Medicine, Monash Medical Centre, Clayton, VIC 3168, Australia; ^2^Department of Nephrology, Monash Health, Clayton, VIC 3168, Australia

## Abstract

Neutrophils are no longer seen as leukocytes with a sole function of being the essential first responders in the removal of pathogens at sites of infection. Being armed with numerous pro- and anti-inflammatory mediators, these phagocytes can also contribute to the development of various autoimmune diseases and can positively or negatively regulate the generation of adaptive immune responses. In this review, we will discuss how myeloperoxidase, the most abundant neutrophil granule protein, plays a key role in the various functions of neutrophils in innate and adaptive immunity.

## 1. Neutrophils in Innate and Adaptive Immunity: The Role of MPO

Neutrophils are capable of affecting many aspects of both innate and adaptive immunity. They are well known to be the first leukocyte to arrive at sites of infection. There, they play a key role in the clearance of pathogens, both by phagocytosis and by subsequent intracellular killing, as well as the release of neutrophil extracellular traps (NETs) into the extracellular space [1]. However, through the release of various inflammatory mediators, neutrophils can also contribute to tissue injury and organ damage in inflammatory and autoimmune diseases. On the other hand, neutrophil proteins are targets in autoimmune anti-neutrophil cytoplasmic antibody- (ANCA-) associated vasculitis (AAV) [2]. In more recent years, evidence has been accumulating to show that not only do neutrophils act at sites of inflammation, but they also infiltrate secondary lymphoid organs where they regulate the development of adaptive immunity [3]. MPO, the major protein in neutrophil granules, has been shown to be one of the key players in the neutrophil functions described above. This paper will review the contribution of MPO to neutrophil-mediated intracellular microbial killing, formation of NETs, and tissue damage, as well as the development of AAV. Particular attention will be given to the more recently described and less well known function of neutrophil MPO as a regulator of adaptive immunity.

## 2. Biosynthesis, Cellular Sources, Storage, and Release of MPO

MPO, which was originally named verdoperoxidase due to its intense green colour, is a highly cationic, heme-containing, glycosylated enzyme [4] which is found mainly in primary (azurophilic) granules of neutrophils, making up approximately 5% of the total dry cell weight [5]. Human neutrophils contain about 5–10-fold higher levels of MPO than murine neutrophils [6]. MPO is also found, to a lesser extent, in monocytes where it constitutes about 1% of total cell protein [7]. During monocyte-to-macrophage differentiation, MPO expression is generally lost [8]. However, MPO can be found in some macrophage subpopulations including resident tissue macrophages such as Kupffer cells [9], peritoneal macrophages [10], and microglia [11], as well as in organ infiltrating macrophages in various inflammatory diseases including atherosclerosis [12], multiple sclerosis (MS) [13], and AAV [14]. Macrophages can also acquire neutrophil-derived MPO by phagocytosis of apoptotic neutrophils or uptake of extracellular MPO through the mannose receptor [15].

Although it is possible for MPO transcription to be reinitiated in macrophages under certain conditions [16], MPO synthesis is otherwise restricted to myeloid cells in the bone marrow [17, 18]. During granulocyte/monocyte differentiation in the bone marrow, only promyelocytes and promyelomonocytes actively transcribe the MPO gene [17]. The primary 80 kDa MPO translation product undergoes cleavage of the signal peptide and N-linked glycosylation [17], resulting in a 90 kDa apoproMPO which is heme-free and thus enzymatically inactive [17]. During association with endoplasmic reticulum chaperones, calreticulin and calnexin, apoproMPO acquires heme and becomes the enzymatically active precursor proMPO [19], which then enters the Golgi. After exiting the Golgi, a series of proteolytic steps follow during which the propeptide is removed and the protein is cleaved into a heavy (*α*) subunit (59 kDa) and a light (*β*) subunit (13.5 kDa) joined together by disulfide bonds [8]. This heavy-light chain complex dimerizes to generate mature, enzymatically active MPO containing a pair of heavy-light protomers and two heme groups [8]. Human and mouse MPO have molecular weights of 146 kDa and 135 kDa, respectively [8, 20].

Mature MPO is stored in azurophilic granules of fully differentiated neutrophils. However, following priming and activation by inflammatory mediators including TLR ligands and cytokines such as GM-CSF and TNF, as well as Ig/Fc receptor-mediated signals [22–24], MPO can be released via multiple mechanisms. Neutrophils can rapidly release MPO by degranulation and by cell death pathways including apoptosis and necrosis [8, 21, 25]. More recently, it has been shown that MPO can be released from neutrophils via the extrusion of NETs [26]. Interestingly, some proMPO is also constitutively secreted by neutrophils via the Golgi [17], but the function and physiological relevance of extracellular proMPO are still to be elucidated.

## 3. Production of Reactive Intermediates by MPO

**Figure 1:**
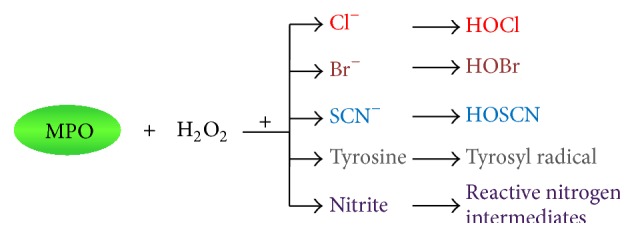
*Reactive intermediates formed by MPO*. In the presence of hydrogen peroxide and chloride, bromide, thiocyanate, tyrosine, or nitrite, MPO catalyses the formation of hypochlorous, hypobromous, and hypothiocyanous acids, tyrosyl radical, and reactive nitrogen intermediates. H_2_O_2_, hydrogen peroxide; Cl^−^, chloride; Br^−^, bromide; SCN^−^, thiocyanate; HOCl, hypochlorous acid; HOBr, hypobromous acid; HOSCN, hypothiocyanous acid.

In the presence of hydrogen peroxide (H_2_O_2_) and a low-molecular-weight intermediate (halide: chloride, bromide, or thiocyanate; tyrosine; or nitrite) MPO catalyses the formation of powerful reactive intermediates including hypochlorous (HOCl), hypobromous (HOBr), and hypothiocyanous (HOSCN) acids, tyrosyl radical, and reactive nitrogen intermediates (Figure 1), all of which can have profound effects on cellular function by modifying proteins, lipids, and/or DNA [8]. The H_2_O_2_ required for MPO function comes mainly from the phagocyte NADPH oxidase during the respiratory burst [8]. Given its abundance in physiological fluids [27], chloride is believed to be the physiological halide and, therefore, a preferred substrate for MPO and subsequent HOCl production in most circumstances.

HOCl is a short-lived, but very potent chlorinating oxidant [28]. It can oxidize/chlorinate a variety of targets including proteins, lipids, and DNA and thus have significant biological effects [28]. Formation of 3-chlorotyrosine (due to HOCl chlorination of tyrosine on proteins) and, more recently described, glutathione sulfonamide (GSA; a product of HOCl-mediated oxidation of glutathione), serves as specific biomarkers of MPO/HOCl production* in vivo* [8, 28–30]. Taurine, a free amino acid present at high concentrations in neutrophils [31], also readily reacts with HOCl to form taurine chloramine, a less reactive, but long-lived oxidant which can contribute to cell damage [8]. More detailed descriptions of the reactions catalysed by MPO and the oxidants it produces are provided elsewhere [8, 32].

## 4. The MPO/HOCl System in Intracellular and Extracellular Microbial Killing by Neutrophils

Neutrophils are one of the most important front line defenders involved in microbial ingestion and subsequent killing. Several lines of evidence demonstrate that the MPO/HOCl system plays an important role in optimal intracellular killing of bacteria (e.g.,* Pseudomonas aeruginosa*) and fungi (e.g.,* Candida albicans*) by neutrophils [8, 33, 34]. It should be noted, though, that the clearance of several pathogens including* Staphylococcus aureus* and* Candida glabrata* is not affected by the absence of MPO [8, 34]. In addition, the majority of MPO-deficient patients do not suffer from chronic infections despite the demonstration of a neutrophil microbicidal defect* in vitro* [8]. This suggests the existence of MPO-independent antimicrobial systems such as reactive nitrogen intermediates and proteases which have been shown to contribute to microbicidal activity of neutrophils in the presence as well as in the absence of MPO [32, 35]. In addition, the reduction of microbial killing due to the absence of MPO in humans may be compensated by an enhancement of protective (i.e., antimicrobial) adaptive immunity, as discussed in more detail below. For in-depth discussion about the well recognised and extensively studied role of MPO in intracellular microbial killing by neutrophils, we suggest references to more comprehensive past reviews [32].

Neutrophils are also well known to release NETs, structures composed of decondensed chromatin, histones, and various antimicrobial molecules including elastase and MPO, into the extracellular space [1], thus aiding in the overall elimination and spread of pathogens. NETs can trap extracellular microbes that they come in contact with and, although limited, evidence also exists to suggest that NETs can kill some, but not all, extracellular microbes [36, 37]. Together with elastase, MPO has been demonstrated to associate with nuclear DNA/histones and play a role in NET formation as well as NET-mediated bacterial killing [38–40]. Studies with normal and MPO-deficient human neutrophils showed that MPO contributes to the formation/release of NETs in response to stimulation with PMA or* Candida albicans* [38]. Similarly, the induction of NETs in neutrophils from healthy donors via TNF, IL-8, or IL-1*β*, in the absence of any infectious stimuli, required the presence of active MPO [41]. A recent study has added to our understanding of how MPO contributes to NET formation inside human neutrophils by showing that MPO activates elastase allowing it to enter the nucleus where it can then associate with DNA/histones [42].

However, MPO is not required for NET formation with all stimuli. For example, in human neutrophils stimulated with* S. aureus* or* E. coli*, inhibition of MPO had no effect on NETs [43]. Reports in which human neutrophils were stimulated with* Pseudomonas aeruginosa* have yielded conflicting results [43, 44], the explanation for which is still to be provided. Furthermore, studies using cells from MPO-deficient animals or inhibitors of MPO activity showed that MPO is not involved in PMA/bacteria-induced NETosis by murine neutrophils [44]. Mouse neutrophils do contain less MPO than their human counterparts [6], which may provide a partial explanation for the discrepancy between the human and murine studies.

In addition to playing a role in NET formation, MPO has been demonstrated to contribute to NET-mediated killing of extracellular microbes. NET-associated MPO is enzymatically active and can produce HOCl in the presence of its substrate, H_2_O_2_ [39]. Production of HOCl by MPO bound to NETs resulted in the killing of* S. aureus in vitro* and inhibition of MPO or addition of a strong HOCl scavenger methionine reversed this effect [39]. This suggests that HOCl generated by NET-associated MPO may also play an important role in extracellular bacterial killing* in vivo* at sites of inflammation.

## 5. MPO-Derived Oxidants Cause Tissue Injury in Inflammatory/Autoimmune Diseases

Through the release of various mediators including reactive oxygen species (ROS), proinflammatory cytokines, and proteases, neutrophils play an important role as effector cells in many inflammatory and autoimmune diseases including cardiovascular disease and atherosclerosis, rheumatoid arthritis (RA), and inflammatory diseases of the lung and kidney [1]. Through the formation of reactive oxidating/chlorinating agents, MPO is one of the key neutrophil-derived mediators contributing to organ inflammation and fibrosis in many immune-mediated diseases. As this classical effector function of MPO has been comprehensively described in many other reviews [8, 45], it is not discussed here in detail. Active MPO and/or its products such as HOCl-modified proteins, 3-chlorotyrosine and GSA, are upregulated at sites of inflammation in cardiovascular disease and atherosclerotic lesions, RA joints, and lungs of patients with cystic fibrosis and inflammatory and fibrotic kidney disease [12, 29, 46–49]. This indicates MPO-mediated damage by reactive oxidants, mainly HOCl. Reports showing that there is significant attenuation or exacerbation of disease due to the absence of endogenous or administration of exogenous MPO, respectively, in models of these conditions [50–58], further support the hypothesis that MPO is an important local mediator of inflammation and subsequent organ damage.

MPO-containing NETs have also been implicated in the pathogenesis of several inflammatory diseases including systemic lupus erythematosus (SLE), atherosclerosis, and RA. For example, glomerular NET deposition positively correlates with anti-dsDNA autoantibody levels and the severity of lupus nephritis [59]. Recently, NET-induced macrophage activation and cytokine production have been reported to contribute to the development of atherosclerotic lesions [60]. In addition, lipid oxidation/chlorination by the MPO/HOCl system is suggested to contribute to the pathogenesis of atherosclerosis and SLE [61, 62]. Enhanced formation of NETs, providing a source of citrullinated autoantigens, is also observed in joints of patients with RA [63]. MPO is present on NETs in target organs in SLE, atherosclerosis, and renal vasculitis [14, 59, 60] and it is therefore plausible, although not yet experimentally confirmed, that NET-bound MPO contributes to organ inflammation and injury in those conditions given that NET-associated MPO is enzymatically active and can produce tissue damaging HOCl in the presence of H_2_O_2_ [39].

## 6. MPO as an Autoantigen in AAV

In addition to neutrophil-derived inflammatory mediators contributing to tissue damage in inflammatory conditions as effector molecules, neutrophil proteins such as proteinase-3 and MPO play a key role in the development of autoimmune AAV, acting as targets (i.e., autoantigens) against which the pathogenic immune response has been generated. MPO is a common autoantigen in AAV, a disease characterised by inflammation of small blood vessels including glomerular capillaries in the kidney and commonly associated with the presence of pathogenic neutrophil-activating MPO-ANCA [64]. Renal biopsies from vasculitis patients show a prominence of glomerular delayed-type hypersensitivity effectors (T cells, macrophages, and fibrin) and neutrophils, suggesting that cellular immunity, together with MPO-ANCA, plays a significant part in the disease process [65].

Evidence from animal studies, which is supported by human observations and* in vitro* experiments [65–72], suggests that the pathogenesis of MPO-AAV involves 4 major steps, as outlined below. First, MPO-specific autoimmunity develops in secondary lymphoid organs, resulting in the emergence of autoreactive effector CD4 T cells and MPO-ANCA-producing B cells. Although it is not known how autoimmunity to MPO is generated in humans, evidence from animal studies shows that activation of myeloid DCs by NETotic, but not apoptotic or necrotic, neutrophils can result in the generation of MPO-specific autoimmunity and development of renal vasculitis [73]. Of note, the induction of autoimmunity by NET-activated DCs in the animal studies was not restricted to MPO but also resulted in the generation of anti-dsDNA antibodies [73] which are associated with SLE. This is not surprising since NET release by neutrophils would expose a variety of intracellular autoantigens for presentation to DCs. Recently, the immunodominant MPO T cell epitope (MPO_409–428_) was defined in mice [72], and, interestingly, it showed significant overlap with the dominant B cell epitope in AAV patients [74], indicating its relevance to human disease. Importantly, MPO_409–428_ was shown to be nephritogenic since immunisation of mice with MPO_409–428_ resulted in the generation of pathogenic MPO-specific CD4 T cells and ANCA [72].

Second, neutrophil priming by cytokines (e.g., TNF), which may occur after infection-related stimuli, leads to MPO exposure on the cell surface, allowing ANCA to bind and fully activate the neutrophils. Third, ANCA-activated neutrophils lodge in glomeruli [75], causing injury [76] and depositing the autoantigen, MPO [69]. Finally, MPO-specific effector CD4 T cells migrate to inflamed glomeruli where they recognise MPO and direct accumulation of macrophages and fibrin, causing, together with ANCA-induced neutrophil responses, severe and proliferative renal vasculitis (glomerulonephritis; GN) [69, 72]. The antigen presenting cells exposing MPO peptides/MHC-II for recognition by effector CD4 T cells in glomeruli are yet to be identified; however several potential candidates exist including intrinsic glomerular cells such as endothelial cells and podocytes, as well as kidney-infiltrating MPO-positive macrophages and neutrophils. All these cell types can upregulate surface MHC-II and costimulatory molecules in response to proinflammatory stimuli and have been shown to contain MPO in biopsies from patients with AAV [14, 77–80].

In addition to acting as an autoantigen in AAV, MPO may contribute to disease pathogenesis through its enzymatic activity and production of oxidative/chlorinating radicals, although this remains to be confirmed. Extracellular, including NET-associated, MPO is pronounced in glomeruli of AAV patients [14, 26]. Future studies are yet to demonstrate the presence of specific biomarkers of MPO activity such as 3-chlorotyrosine, HOCl-modified proteins, or GSA, to suggest MPO-mediated damage in AAV. Moreover, recent advancements in the development of specific MPO inhibitors for* in vivo* use [81–83] should make it more feasible to investigate whether MPO contributes to renal injury in models of AAV via its enzymatic activity.

## 7. Nonenzymatic Functions of MPO in Inflammation

MPO actions are mediated predominantly via its enzymatic activity and generation of reactive intermediates. However, evidence exists demonstrating that it can also regulate the function of immune and nonimmune cells via its nonenzymatic effects. For example, by binding to CD11b/CD18 (Mac-1), MPO can induce neutrophil activation in an autocrine fashion including MAPK and NF*κ*B activation, ROS production, surface integrin upregulation, and degranulation [84], as well as decreased apoptosis leading to enhanced inflammation in the lung [85]. In addition, human leukocytes can adhere to MPO via binding to CD11b/CD18 [86] which may also contribute to the proinflammatory effects of MPO by further augmenting leukocyte accumulation at sites of inflammation. Inactive MPO has also been shown to increase macrophage activation such as cytokine production and induction of respiratory burst* in vitro* [87]. These observations are likely to be relevant* in vivo* as well since injection of inactivated MPO into the joints of rats exacerbated symptoms of arthritis [52]. Here, the proinflammatory effects of MPO were reversed by injection of mannan, thus most likely blocking the interaction between extracellular MPO and mannose receptor on macrophages [52]. Furthermore, enzymatically inactive MPO can activate endothelial cells to produce cytokines such as IL-6 and IL-8 [88]. The exact mechanisms by which this occurs are unknown, but MPO-mediated endothelial cell activation is likely to add to the proinflammatory effects of MPO, since the leukocyte-endothelial cell interaction is one of the critical processes in inflammatory responses within tissues.

## 8. MPO Suppresses the Generation of Adaptive Immunity

In addition to playing an important role at sites of inflammation, neutrophils have been shown to contribute to the development of adaptive immunity. A number of studies have shown that neutrophils can attract and activate immature DCs at sites of inflammation, as well as promote DC trafficking to draining lymph nodes, thus augmenting adaptive immune responses [89–92]. Neutrophils can also rapidly migrate to lymph nodes after antigen injection, mainly via the lymphatics, in a CD11b-, CXCR4-, and, in some cases, CCR7-dependent manner, where they either enhance or suppress the subsequent induction of T cell responses [3, 92–94]. Neutrophil-mediated inhibition of adaptive immunity in mice is supported by studies in humans showing that a subset of human neutrophils can attenuate T cell responses [95]. However, the mechanisms and mediators by which neutrophils inhibit the generation of adaptive immunity in lymph nodes are not well known. Recent studies demonstrating that MPO plays a key role in neutrophil-mediated suppression of adaptive immunity have provided important insights into these important, but underexplored issues [21, 56, 57, 96].

**Figure 2:**
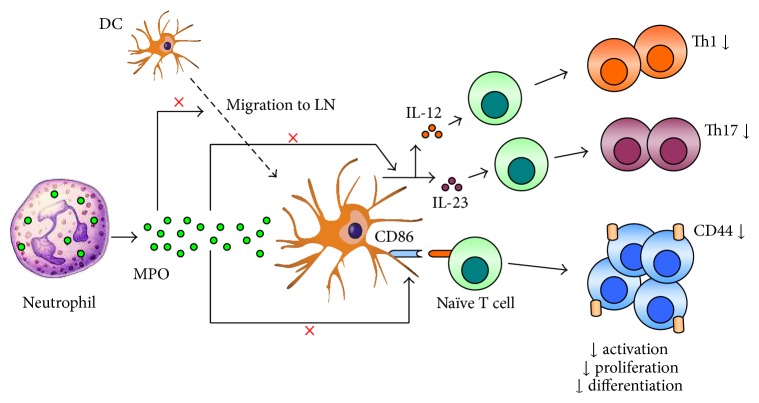
*Neutrophil MPO suppresses DC function and adaptive immunity*. Odobasic et al. [21] showed that rapidly infiltrating neutrophils release MPO in draining lymph nodes (LN) after antigen/adjuvant injection. The deposited MPO suppresses various aspects of DC function including costimulatory molecule (e.g., CD86) expression and cytokine (IL-12, IL-23) production and migration, resulting in decreased generation of CD4 T cell responses including T cell activation (CD44 expression), proliferation, and differentiation into Th1 (IFN*γ*-producing) and Th17 (IL-17A-releasing) effectors.

Our group has shown that neutrophils rapidly and transiently infiltrate draining lymph nodes after antigen injection [21], as observed in other models [97]. Four hours after OVA/LPS injection, neutrophils degranulated and deposited MPO in lymph nodes, where it interacted with DCs [21], suggesting that extracellular MPO may affect DC function and subsequent induction of adaptive immunity. Further experiments using MPO-deficient mice or a specific MPO inhibitor, 4-aminobenzoic acid hydrazide (ABAH), showed that MPO, via its enzymatic activity, suppresses various aspects of DC function* in vivo* (Figure 2) including their activation (costimulatory molecule and MHC-II expression, cytokine production), antigen uptake/processing and migration to lymph nodes (by decreasing CCR7 expression), without affecting their apoptosis [21]. MPO-mediated suppression of DC function correlated with decreased generation of adaptive CD4 T cell (particularly Th1) immunity [21]. The suppressive effects of MPO on DCs were confirmed* in vitro* by culturing bone marrow-derived DCs with LPS and either supernatant from wild type (WT) or MPO−/− neutrophils degranulated in the presence or absence of ABAH, or purified enzymatically active native mouse MPO with or without ABAH. Importantly,* in vitro* studies using monocyte-derived DCs and supernatant from degranulated human neutrophils (±ABAH) or purified human MPO showed that MPO has similar inhibitory effects on DCs in humans [21]. Further mechanistic experiments indicated that HOCl and, to a lesser degree, HOBr are the main products involved in MPO-mediated suppression of DC activation* in vitro* [21], consistent with previous studies showing that taurine chloramine (a product formed by the reaction of HOCl and the amino acid taurine) can decrease DC maturation [98]. HOSCN also had inhibitory effects on DCs, but to a much lesser extent than HOCl and HOBr [21], consistent with HOSCN being a much less reactive oxidant [99]. In contrast, MPO-mediated consumption of nitric oxide, which itself can reduce DC maturation [100], reversed the effects of HOCl on DC activation* in vitro*. Interestingly, Mac-1, an inhibitory receptor on DCs [101], was shown to be involved in the enzymatic MPO-mediated suppression of DC IL-12 production [21]. Although the exact pathways involved in this process are still to be identified, these observations may be explained, in part, by previous reports showing that oxidants (thus potentially MPO-derived products) can induce activating conformational changes in Mac-1 [102], which would be expected to inhibit DC function.

The inhibition of DC function and subsequent generation of adaptive immunity by MPO are relevant to immune-driven diseases since it can result in attenuation of certain T cell-mediated inflammatory conditions. For example, in a model of lupus nephritis, dependent on both autoreactive T cells and humoral immunity [103, 104], we showed that MPO-deficient mice develop more severe renal injury in association with enhanced accumulation of cellular effectors, CD4 T cells, macrophages, and neutrophils [96]. This in turn correlated with enhanced activation of DCs and increased T cell autoimmunity in lymph nodes and spleen. Of note, augmented renal injury was observed in MPO−/− mice despite reduced deposition of humoral mediators of injury (antibody and complement) in glomeruli and decreased presence of markers of oxidative damage, 8-hydroxydeoxyguanosine and GSA [96]. Therefore, in experimental lupus nephritis, MPO-mediated suppression of pathogenic T cell autoimmunity overrides the local damaging effects of MPO in the kidney. These results are concordant with observations in humans showing an increased incidence of lupus nephritis in patients with a polymorphism causing reduced MPO expression [105]. Similarly, in antigen-induced arthritis (AIA), which is very T cell-driven [106], MPO−/− mice developed more severe joint inflammation and damage in association with augmented CD4 T cell responses in the spleen [21]. Furthermore, Brennan et al. demonstrated that MPO−/− mice develop more severe disease in experimental autoimmune encephalomyelitis (EAE), a model of MS, correlating with higher antigen-specific lymphocyte proliferation in draining lymph nodes [13]. Collectively, these studies are supported by a report showing increased incidence of chronic inflammatory conditions in MPO-deficient patients [107], indicating their relevance to humans.

In another murine model of GN induced by a planted foreign antigen (sheep globulin) against the glomerular basement membrane (GBM), our group reported that MPO−/− mice are protected from renal injury in the initial (heterologous) phase of the disease [56] which is mediated by neutrophils but is independent of T cells [108], showing that neutrophil-derived MPO contributes to kidney damage locally. However, during the later (autologous) T cell/macrophage-mediated phase of the disease [109, 110], renal injury was similar between WT and MPO−/− mice, despite enhanced adaptive immunity in the spleen and increased glomerular accumulation of T cells and macrophages in MPO-deficient animals [56]. Together, these experiments suggested that the inhibitory effects of MPO on adaptive immunity in secondary lymphoid organs can also be counterbalanced by the local pathogenic effects of MPO in the target organ.

In other inflammatory conditions though, the injurious local effects of MPO can dominate over its inhibitory effects on immune responses in lymph nodes and spleen, leading to exacerbation of disease, as shown in some models of RA. For example, in collagen-induced arthritis (CIA), mediated by autoreactive T cells and antibody, but also neutrophils [111–113], we demonstrated that disease is attenuated due to MPO deficiency despite enhanced T cell autoimmunity in secondary lymphoid tissues, without an effect on autoantibody levels [57]. This suggested that MPO has dominant proinflammatory local effects in the joints which was confirmed in an acute neutrophil-mediated, T cell-independent KB × N model [114] by showing that joint inflammation and damage were significantly reduced in MPO−/− mice without an effect on circulating cytokines [57].

Overall, these studies indicate that the net impact of MPO on disease development depends on the balance between its local injurious effects in the target organ and inhibitory effects on adaptive immunity in secondary lymphoid tissue and that this balance varies in different autoimmune diseases. Although it is not clearly understood which factors tip this balance in either direction, the above studies do suggest that under conditions where neutrophils play a very important role as effectors of injury in the inflamed organs (e.g., CIA, KB × N arthritis, heterologous anti-GBM GN), the local pathogenic effects of MPO predominate. In contrast, in situations where T cell immunity is the main driver of disease with lesser involvement of neutrophils as effectors (e.g., AIA, lupus nephritis, autologous anti-GBM GN, and EAE), the immunosuppressive effects of MPO in secondary lymphoid organs predominate, tipping the balance towards MPO-mediated attenuation of disease.

## 9. MPO Deficiency in Humans

Hereditary MPO deficiency in humans is not rare, with reported prevalence in the United States and Europe ranging from 1 : 1000 to 1 : 4000 [7, 115–118]. Patients lacking MPO are more susceptible to fungal infections, particularly those caused by* Candida albicans* [118–120]. This is in line with murine studies [33] and the MPO/HOCl system being critical for the direct killing of the fungus and* C. albicans*-induced NET formation [38, 53, 118]. Although reports exist demonstrating that MPO-deficient patients can have a higher incidence of severe infections [107, 121], the majority of patients lacking MPO have been shown not to be particularly susceptible to chronic infections [8]. Similar to humans, MPO knockout mice exhibit higher susceptibility to some, but not all infections including those caused by* Candida glabrata*,* S. aureus*, and* S. pneumoniae* [8, 34]. This may be due to several factors: (i) MPO-deficiency increases the expression and activity of inducible NO synthase resulting in augmented levels of NO and reactive nitrogen intermediates which have been shown to play a role in the killing of microbes by neutrophils in the presence and in the absence of MPO [32, 35], (ii) the lack of the MPO/HOCl system results in increased activity of antimicrobial neutrophil granule proteases such as elastase and cathepsin-G [122–124], (iii) neutrophils from MPO-deficient patients have increased phagocytosis and degranulation [125, 126], and (iv) the absence of MPO augments the generation of adaptive immunity, as shown by us and others [13, 21, 56, 96]. Therefore, decreased microbial killing due to the absence of MPO/HOCl may be compensated for by other systems involved in pathogen clearance, including increased expression reactive nitrogen intermediates, enhanced activity and release of proteases from neutrophil granules, augmented phagocytic activity of neutrophils, and increased adaptive immunity against the invading microbes.

Although the clinical consequences of MPO deficiency in humans have not been thoroughly investigated, some studies have found that patients with total or subtotal lack of MPO have an increased incidence of chronic inflammatory conditions [107, 121] which are known to be mediated by the adaptive immune system. Similarly, patients with a genetic polymorphism resulting in decreased expression of MPO have a higher risk of developing autoimmune lupus nephritis [105], and MS and diabetes patients have been reported to have lower MPO activity in their blood leukocytes [127, 128]. These observations may be, in part, explained by studies showing increased development of adaptive immune responses and T cell-driven inflammatory conditions in MPO-deficient animals [13, 21, 56, 96], as discussed above.

## 10. Conclusion

**Figure 3:**
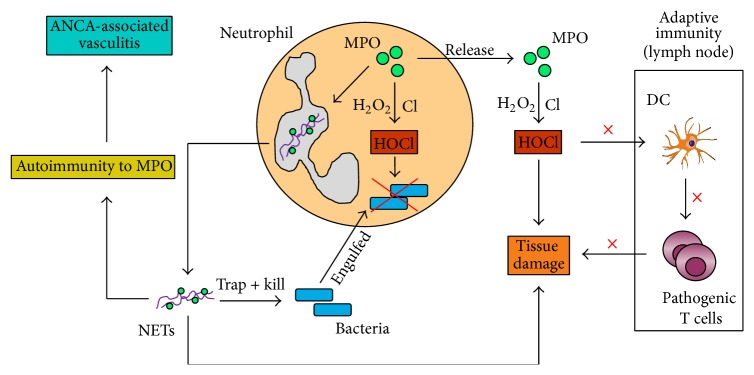
*Summary of MPO involvement in neutrophil functions in innate and adaptive immunity*. MPO is involved in microbe clearance by neutrophils both intracellularly (via the production of HOCl) and extracellularly (via the release of NETs). On the other hand, the release of MPO-containing NETs can result in the generation of autoimmunity against MPO and subsequent development of ANCA-associated vasculitis. HOCl that is produced outside of activated neutrophils following MPO release can cause significant tissue damage. In contrast, MPO that is released by neutrophils in lymph nodes can inhibit DC activation and thus generation of adaptive T cell responses, thus attenuating organ injury. HOCl, hypochlorous acid; H_2_O_2_, hydrogen peroxide; Cl, chloride; NETs, neutrophil extracellular traps; DC, dendritic cell; ANCA, anti-neutrophil cytoplasmic antibody.

Neutrophils use MPO to mediate many of their multifaceted functions that they have in the immune system (summarised in Figure 3). Through the production of HOCl in the presence of H_2_O_2_ and chloride, MPO plays an important role in the killing of microbes which have been engulfed by neutrophils. MPO can also associate with nuclear DNA/histones and contribute to the formation/release of NETs which trap and sometimes kill (via MPO-produced HOCl) extracellular bacteria. On the other hand, stimulation of DCs by NET-bound MPO can result in the generation of MPO-specific autoimmunity and subsequent development of AAV. MPO release and subsequent formation of HOCl in the extracellular environment following neutrophil activation have been shown to contribute to tissue inflammation and damage. Importantly, MPO deposited by neutrophils in lymph nodes can inhibit DC activation and subsequent generation of adaptive T cell immunity thus leading to attenuation of immune-mediated tissue injury. Future studies will not only further our understanding about these already described functions of MPO but are also likely to uncover novel roles of this neutrophil enzyme in the regulation of cellular events that take place during the course of innate and adaptive immune responses.
